# Two Siblings With Microcephalic Osteodysplastic Primordial Dwarfism Type II (MOPDII) Caused by Compound Heterozygous Pericentrin (PCNT) Gene Variants

**DOI:** 10.7759/cureus.99990

**Published:** 2025-12-24

**Authors:** Ahmed Al Farsi, Lina Abdullah, Amr Khalil, Tarek El-Alem

**Affiliations:** 1 Pediatrics, NICU, London Health Sciences Centre, London, CAN; 2 Pediatrics, NICU, Hamad Medical Corporation, Doha, QAT; 3 Pediatrics, NICU, Sidra Medicine, Doha, QAT

**Keywords:** compound heterozygous, intrauterine growth restriction, microcephalic osteodysplastic primordial dwarfism type ii, microcephaly, mopdii, pcnt gene, pericentrin, prenatal diagnosis, primordial dwarfism, skeletal dysplasia

## Abstract

Microcephalic osteodysplastic primordial dwarfism type II (MOPDII) is a rare genetic disorder characterized by severe pre- and postnatal growth failure and microcephaly. We present two male siblings with MOPDII confirmed by the identification of compound heterozygous variants in the pericentrin (PCNT) gene. The first, a twin with intrauterine growth restriction, was diagnosed postnatally at four years of age after developing dysmorphic features and neurodevelopmental delays. His younger sibling was diagnosed prenatally via chorionic villus sampling. These cases highlight that MOPDII can result from compound heterozygous inheritance and underscore the importance of considering this diagnosis in severe, symmetric intrauterine growth restriction. A structured, multidisciplinary approach is essential for managing the complex comorbidities, including the high risk of cerebrovascular disease.

## Introduction

Microcephalic osteodysplastic primordial dwarfism type II (MOPDII) is the most common form of microcephalic primordial dwarfism, a group of rare autosomal recessive disorders characterized by extreme prenatal and postnatal growth restriction [1]. First described by Majewski et al. in 1982 [2], MOPDII was later linked to biallelic loss-of-function mutations in the pericentrin (PCNT) gene [3]. Although the exact prevalence is unknown, it is an exceedingly rare condition, with more than 150 molecularly confirmed cases reported worldwide [1].

The PCNT gene encodes pericentrin, a large scaffolding protein that is an integral component of the centrosome. The centrosome functions as the primary microtubule-organizing center in animal cells, and pericentrin is essential for anchoring a multitude of regulatory proteins and protein complexes to it [4]. Through these interactions, pericentrin plays a critical role in fundamental cellular processes, including cell cycle progression, the establishment of mitotic spindles, and the maintenance of genomic stability [4,5].

The pathogenesis of microcephalic osteodysplastic primordial dwarfism type II (MOPDII) is attributed to pericentrin dysfunction, resulting in the disruption of centrosome integrity and profound mitotic defects. Experimental and mechanistic studies have shown that loss of functional PCNT impairs centrosomal anchoring of key regulatory proteins, including checkpoint proteins, such as Chk1, leading to dysregulated cell-cycle control and abnormal mitotic entry. In addition, defective recruitment of γ-tubulin complexes compromises microtubule nucleation, resulting in disorganized mitotic spindles and chromosome segregation errors. Collectively, these centrosome-related defects are thought to reduce cellular proliferation during development, contributing to tissue hypoplasia, growth failure, and microcephaly [4,5].

The clinical phenotype of MOPDII is complex and extends beyond its defining features of microcephaly and dwarfism. The diagnostic journey typically begins with the recognition of severe intrauterine growth restriction (IUGR) and profound postnatal growth failure. While the age of diagnosis varies, it often occurs in early childhood. Radiographic imaging reveals a characteristic skeletal dysplasia, including delayed bone age and hip abnormalities. However, what distinguishes MOPDII and contributes significantly to its morbidity and mortality is the presence of a global vascular disease, including a high risk of cerebrovascular complications like moyamoya disease and intracranial aneurysms, as well as coronary artery disease [6]. Furthermore, metabolic complications, such as insulin resistance and diabetes mellitus, are common [7]. The definitive diagnosis is established through molecular genetic testing that identifies biallelic loss-of-function variants in PCNT.

The natural history of MOPDII is characterized by high childhood and juvenile mortality and markedly reduced survival, with premature death most commonly resulting from cerebrovascular events, cardiac complications, and systemic metabolic derangements [8]. Early recognition of MOPDII is crucial, as it allows for the implementation of proactive surveillance protocols aimed at detecting and managing life-threatening complications, particularly cerebrovascular disease, which necessitates regular neuroimaging and multidisciplinary care [1,9]. Due to the condition’s rarity, each new case report is valuable for defining the full clinical spectrum and refining management strategies.

We present a case series of two male siblings with MOPDII that contributes to the literature in several important ways. This report highlights the value of early diagnosis by contrasting a postnatal diagnosis in the first sibling with a prenatal diagnosis in the second, which allowed for immediate implementation of a surveillance protocol. Furthermore, it underscores the genetic heterogeneity of MOPDII. The affected siblings harbor compound heterozygous PCNT variants, inheriting a different pathogenic allele from each carrier parent. While homozygous mutations are often expected in autosomal recessive disorders, particularly in consanguineous families, the observation of compound heterozygosity is frequent in MOPDII and reflects the diverse mutational spectrum of the PCNT gene [3,10]. This report, therefore, provides valuable insights into clinical management, genetic counseling, and the molecular basis of MOPDII.

## Case presentation

Case 1: postnatal diagnosis

Perinatal and Infancy Period

The first case was a male infant, the second twin from a non-consanguineous family, born at 36 weeks of gestation. The pregnancy was complicated by intrauterine growth restriction (IUGR). At birth, his weight, length, and head circumference were all documented to be below the third percentile, and the initial physical examination revealed severe symmetric growth restriction and a unilateral undescended testis. He was subsequently managed in the neonatal unit for low birth weight and poor feeding. Throughout early childhood, he exhibited persistent growth failure, with all growth parameters remaining consistently below the third percentile.

Childhood Clinical Course and Diagnosis

At approximately 2.5 years of age, the patient’s clinical course became more complex with the emergence of neurodevelopmental challenges, including hyperactivity and attention deficits. These were followed by the onset of speech delay and cognitive impairment. During this period, dysmorphic features, including synophrys and a broad nasal tip, became more apparent. A comprehensive workup for his persistent growth failure, including endocrine and metabolic panels, was unremarkable. A skeletal survey performed at 3.5 years of age showed a delayed bone age, hypoplastic iliac bones, and bilateral coxa vara. An abdominal ultrasound revealed a low-lying, malrotated right kidney. The diagnosis of MOPDII was ultimately confirmed at four years of age through whole-exome sequencing, which identified compound heterozygous variants in the PCNT gene. Subsequent parental testing confirmed that each parent was a carrier for one of the pathogenic variants.

Case 2: prenatal diagnosis

Prenatal and Perinatal Period

Five years after the birth of the first affected sibling, the mother conceived again. Given the established diagnosis in the family, prenatal genetic testing was performed. Chorionic villus sampling at 12 weeks of gestation confirmed that the male fetus carried the same compound heterozygous PCNT variants identified in his older brother. The infant was born at 37 weeks of gestation, and all birth measurements (weight, length, and head circumference) again fell below the third percentile. The neonatal examination revealed subtle dysmorphic features consistent with MOPDII, including microcephaly and a prominent nose with a full nasal tip. He was managed in the neonatal unit for low birth weight and was discharged with a comprehensive, multidisciplinary follow-up plan already in place.

Postnatal Investigations

Baseline postnatal investigations were performed to screen for known MOPDII-associated complications. A skeletal survey and a brain MRI/MRA performed at birth were both normal, providing a baseline for future surveillance. An echocardiogram revealed a patent foramen ovale.

## Discussion

This case series adds to the MOPDII literature by documenting a less common inheritance pattern, compound heterozygosity, in a non-consanguineous family. The observation of compound heterozygous PCNT variants aligns with the findings from a foundational study by Rauch et al., which first identified both homozygous and compound heterozygous mutations as the cause of MOPDII [3]. Although homozygous mutations are more frequently observed in autosomal recessive disorders, particularly in consanguineous populations, subsequent molecular analyses have confirmed that compound heterozygosity is common in MOPDII, reflecting allelic heterogeneity of the PCNT gene [10]. Our report reinforces the importance of considering this inheritance pattern in non-consanguineous families presenting with features of primordial dwarfism.

Our findings also highlight the evolving nature of the MOPDII phenotype and the diagnostic challenges it presents. In Case 1, the characteristic dysmorphic and skeletal features became more pronounced over time, a clinical observation that underscores the dynamic progression of the disorder. The initial skeletal survey at birth was normal, which is critical; this finding demonstrates that a normal early skeletal survey does not rule out MOPDII and that longitudinal follow-up is essential for infants with unexplained severe IUGR. This aligns with the natural history described by Hall et al., which documents significant intra- and interfamilial variability in the clinical presentation of MOPDII [8]. The delayed bone age and specific skeletal abnormalities (hypoplastic iliac bones, coxa vara) observed later in childhood in Case 1 are classic radiographic features of MOPDII, but their delayed appearance in our patient provides a valuable clinical pearl for diagnosis.

While genotype-phenotype correlations in MOPDII remain poorly defined, our cases contribute to the broader understanding of the condition’s clinical spectrum [1]. The majority of pathogenic PCNT variants lead to loss or marked reduction in functional pericentrin, thereby causing the core features of the disorder. However, the significant clinical variability reported in the literature suggests that additional genetic or environmental factors may act as phenotypic modifiers. For instance, some reports have linked specific PCNT variants to attenuated growth restriction or additional features, such as central precocious puberty [11,12]. Although the specific variants in our patients were not detailed, the presence of a malrotated kidney in Case 1 is a less commonly reported finding and may broaden the phenotypic spectrum of renal anomalies described in MOPDII.

The management of MOPDII is centered on a proactive, lifelong surveillance program designed to mitigate its severe systemic comorbidities. As outlined in our proposed management table, adapted from established guidelines, the approach includes regular screening for cerebrovascular disease, insulin resistance, hypertension, and skeletal complications (Table 1) [7]. The contrasting diagnostic journeys of the two siblings in our report provide a powerful illustration of the value of this approach. The early prenatal diagnosis in Case 2 allowed for the immediate implementation of a comprehensive surveillance protocol from birth. This stands in stark contrast to the diagnostic odyssey of Case 1 and highlights the profound clinical benefit of early genetic diagnosis in families with a known history of MOPDII. As the literature on long-term outcomes and management remains limited, detailed case reports like this one are crucial for refining best practices and improving the standard of care for individuals with this rare and complex disorder.

**Table 1:** Recommended management and surveillance protocol for MOPDII. DEXA: dual-energy X-ray absorptiometry; CBC: complete blood count; TSH: thyroid-stimulating hormone; T4: free thyroxine; HbA1c: hemoglobin A1c; MRI: magnetic resonance imaging; MRA: magnetic resonance angiography; BUN: blood urea nitrogen; IgG: immunoglobulin G; IgA: immunoglobulin A; IgM: immunoglobulin M; MOPDII: microcephalic osteodysplastic primordial dwarfism type II

System/domain	At diagnosis	Annual screening	Screening from age 5 years
Growth and development	Measure height, weight, head circumference, and plot the results on growth charts. Assess developmental milestones.	Monitor growth parameters. Developmental assessment.	Continue growth monitoring. Educational support assessment.
Skeletal system	Full skeletal survey including spine radiographs. Assess for hip dysplasia, scoliosis, and kyphosis.	Spine radiographs for scoliosis. Hip examination.	Spine radiographs. Bone density scan (DEXA). Orthopedic consultation as needed.
Hematologic system	Complete blood count with differential. Bone marrow evaluation if cytopenias present.	Complete blood count.	Complete blood count. Bone marrow biopsy if indicated.
Endocrine system	Thyroid function tests (TSH, free T4). Fasting glucose and HbA1c. Growth hormone assessment.	Thyroid function tests. Fasting glucose and HbA1c. Lipid profile.	Thyroid function tests. Diabetes screening (glucose, HbA1c). Lipid profile. Pubertal assessment.
Cardiovascular system	Echocardiography. Blood pressure measurement.	Blood pressure monitoring. Lipid profile.	Echocardiography every 2-3 years. Blood pressure monitoring. Lipid profile.
Neurologic system	Brain MRI to assess for aneurysms and vascular anomalies. Developmental assessment.	Neurological examination. Vision and hearing assessment.	Brain MRI or MRA every 3-5 years for aneurysm surveillance. Neurological examination.
Ophthalmologic system	Comprehensive eye examination, including retinal assessment.	Eye examination by ophthalmologist.	Annual eye examination. Retinal imaging if indicated.
Renal system	Renal ultrasound. Serum creatinine and electrolytes. Urinalysis.	Renal function tests (creatinine, BUN). Urinalysis. Blood pressure.	Renal ultrasound every 2-3 years. Renal function tests. Urinalysis.
Dental and oral health	Dental examination. Assessment for dental crowding and malocclusion.	Dental examination every 6 months.	Dental examination every 6 months. Orthodontic evaluation.
Immunologic system	Immunoglobulin levels (IgG, IgA, IgM). Lymphocyte subsets if recurrent infections.	Monitor for infections. Repeat immunology workup if indicated.	Immunologic assessment if recurrent infections occur.
Nutritional assessment	Nutritional evaluation. Caloric intake assessment.	Nutritional counseling. Monitor weight gain.	Nutritional support as needed. Assess for feeding difficulties.
Psychosocial support	Family counseling and genetic counseling. Connect with support groups.	Psychosocial assessment. Educational support planning.	Continued psychosocial support. Transition planning for adolescents.

## Conclusions

In conclusion, the findings from this case series support the inclusion of microcephalic osteodysplastic primordial dwarfism type II (MOPDII) in the differential diagnosis for any infant presenting with severe, symmetric intrauterine growth restriction, even in the absence of a known family history or consanguinity, as in our index case. Furthermore, this report highlights the evolving nature of the MOPDII phenotype. As demonstrated in our first case, characteristic dysmorphic and skeletal findings may not be fully apparent in the neonatal period, underscoring that a normal early skeletal survey does not exclude the diagnosis. The identification of compound heterozygous PCNT variants in this non-consanguineous family serves as a crucial reminder that the condition is not caused exclusively by homozygous mutations, a point of significance for genetic counseling and testing strategies. Finally, the contrasting diagnostic journeys of the two siblings powerfully illustrate the clinical value of a proactive, lifelong, and multidisciplinary surveillance program. The prenatal diagnosis in the second case allowed for immediate implementation of such a protocol, emphasizing the importance of early detection in managing the serious comorbidities associated with MOPDII. While the conclusions drawn from this case series provide valuable clinical insights, they are based on the experience of a single family and require validation in larger cohort studies to establish generalizability.
